# A misleading CMV myocarditis during the COVID-19 pandemic: case report

**DOI:** 10.11604/pamj.2020.36.167.23922

**Published:** 2020-07-09

**Authors:** Sara Oualim, Amal Elouarradi, Sara Hafid, Abdelhamid Naitelhou, Mohamed Sabry

**Affiliations:** 1 Department of cardiology, Cheick Khalifa International University Hospital, Mohammed VI University of Health Sciences (UM6SS), Casablanca, Morocco,; 2 Department of internal medecine, Cheick Khalifa International University Hospital, Mohammed VI University of Health Sciences (UM6SS), Casablanca, Morocco

**Keywords:** COVID-19, cytomegalovirus, myocarditis, immunocompetent

## Abstract

Coronavirus disease 2019 (COVID-19) has been reported as the possible cause of acute myocarditis. Myocarditis is an inflammatory heart disease mostly caused by viral infections. Cytomegalovirus (CMV) primary infection is often not suspected as a cause of myocarditis in immune-competent adults. We report the case of a 37-year-old male admitted with fever, cough and dyspnea. Chest CT showed typical ground-glass changes indicative of viral pneumonia. He was tested negative for COVID-19 but had biological markers that made us still suspect it. He had elevated troponin I level (up to 111.5 ng/mL) and diffuse myocardial dyskinesia along with a decreased left ventricular ejection fraction (LVEF). He was diagnosed with CMV myocarditis with cardiac insufficiency and totally recovered without antiviral therapy. During the COVID-19 pandemic patients may develop myocarditis, still every myocarditis is not a COVID infection. Myocarditis linked to CMV infection may be rare, but life-threatening.

## Introduction

Acute viral infections can lead to heart inflammation, resulting in acute myocarditis. A new coronavirus was identified and named Coronavirus disease 2019 (COVID-19) since December 2019. Common symptoms are fever, cough, myalgia, and/or fatigue. Complications include acute respiratory distress syndrome (ARDS) and acute cardiac injury. Cases of COVID-19 complicated with fulminant myocarditis were described. But not every fever is a COVID-19. Here, we describe a previously healthy young man who developed myocarditis and cardiac insufficiency during the COVID-19 pandemic. We were misled by the clinical, radiological and biological aspect and thought it was a COVID-19 related myocarditis. It turned out to be a cytomegalovirus myocarditis, an unusual manifestation of CMV infection with about 18 cases reported to date in the literature.

## Patient and observation

**Figure 1:**
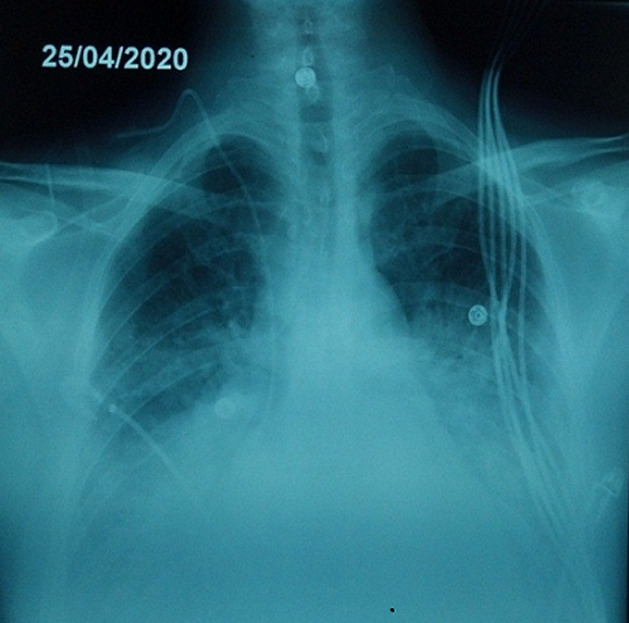
bedside chest radiograph; performed on day 1 showing cardiomegaly, pulmonary edema and small bilateral pulmonary effusions

**Figure 2:**
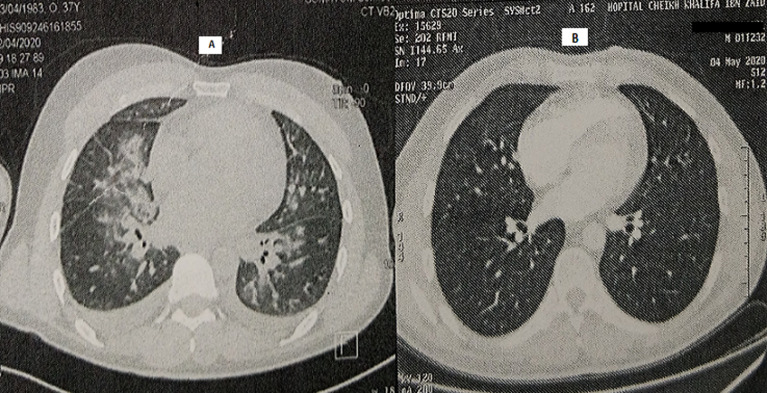
chest computed tomography: (A) obtained on day 1 shows substantial hilar congestion, cardiomegaly, and ground-glass changes; (B) obtained on discharge shows no significant abnormality

**Figure 3:**
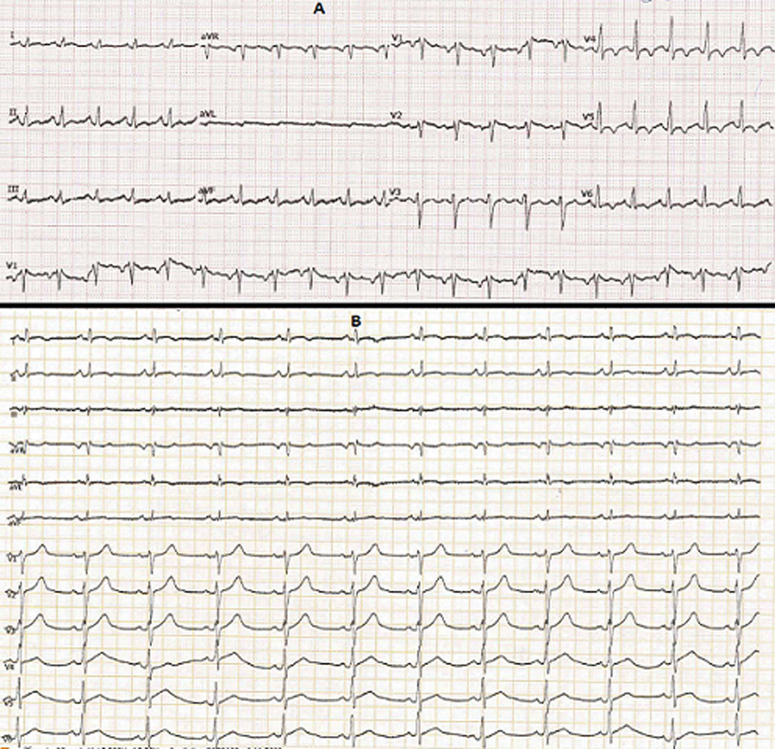
electrocardiogram: (A) on day 1, shows sinus tachycardia at 125 beats/min diffuse and nonspecific disturbances of ventricular repolarization; (B) on discharge showing regression of disturbances of ventricular repolarization

**Figure 4:**
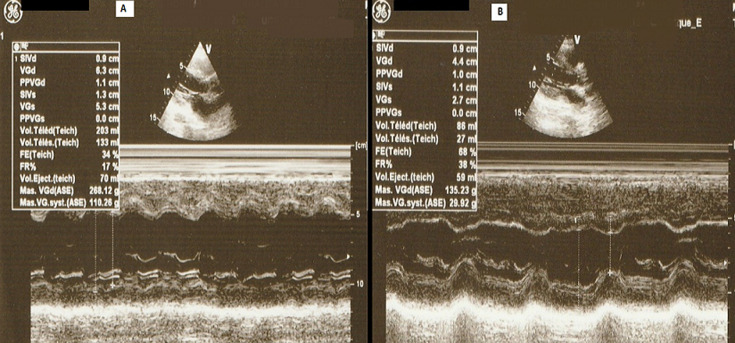
echocardiographic left ventricular M-mode images: (A) Day 1: LV diameter was enlarged, and the ejection fraction was decreased: (B) day 13: normal LVEF (60%) and wall thickness. LV = Left ventricle, LVE = left ventricle ejection fraction

In April 2020, a 37-year-old man was admitted to the emergency department due to the shortness of breath and fever. He complained of severe headaches, myalgias and chest tightness. The patient had fever (39.5°C), orthopnea with arterial oxygen saturation (SaO_2_) at 86% and tachycardia with a heart rate of 125 bpm. Physical examination demonstrated cardiac gallop rhythm and coarse crackles up to the middle third of both lungs. The patient was a non-smoker. He denied alcohol consumption, drug abuse, or recent contact with sick people. The differential diagnosis included pneumonia, heart failure and COVID-19. The chest radiograph revealed para-hilar opacities and enlarged cardiac silhouette (Figure 1). Chest Computed tomography showed ground-glass changes indicative of viral pneumonia (Figure 2A). The electrocardiogram revealed sinus tachycardia, diffuse and nonspecific disturbances of ventricular repolarization (Figure 3A). Laboratory-based tests conducted upon admission revealed the following: hemoglobin level was normal (15.5 g/dL), as well as platelets (296,000/mm^3^), unchanged coagulation; Inflammation syndrome was evident: white cell count was 21390/mm^3^elevated CRP hs (341 mg/L). Blood and urine cultures were all negative. There were increases in lactate dehydrogenase (425 UI/l), ferritin (594 ng/ml) and D-dimer levels (1415 ng/ml). In addition, the patient showed deranged liver and renal function. Markers of myocardial injury included elevated troponin I (Trop I) (111 ng/ml), and n-terminal brain natriuretic peptide (NT-BNP) (1400 pg/ mL). Laboratory data are summarized in Table 1. Two nasopharyngeal swab were taken, one day apart and were negative for COVID-19. We still suspected the COVID-19 as he had many clinical, biological and radiological arguments. Bedside transthoracic echocardiography revealed an enlarged left ventricle (61 mm) (Figure 4A), segmental myocardial hypokinesis along with a low left ventricular ejection fraction (LVEF) (30%) and elevated left ventricular pressures.

**Table 1:** relevant laboratory on admission and subsequent days

Variable	Normal range	Day 1	Day 3	Day 7	Day 13
Hemoglobin (g/dl)	13-18	15	14.8	14.7	15.5
Platelets (10^3^/ mm^3^)	150-400	296	300	369	414
WBC (10^3^ /mm^3^)	4-11	21.39	15.57	8.7	10.26
Neutrophils (10^3^/mm^3^)	1.4-7.7	17.95	10,6	5.3	6.64
Monocytes (/mm^3^)	0.18-1	1.69	1,3	0.9	0.72
CRP hs (mg/l)	< 89	341	307.9	98.4	5.07
Ferritin (ng/ml)	30-300	549	844.88	340	279
LDH (UI/l)	85-230	425	357	277	190
D-dimer (ng/ml)	<500	1415	2083.37	820	486
Troponin I (ng/mL)	<0.03	111	80	30	-
NT-Pro-BNP (pg/ml)	< 300	14000	-	1200	350
AST (U/L)	5-34	108	80	40	37
ALT (U/L)	<55	266	224	106	53
GGT (U/L)	<85	39	40	56	60
ALP (U/L)	40-150	112	103	110	108
Total bilirubin (mg/l)	2-12	24.3	18	5.90	5.26
Creatinine (mg/l)	7-13	14.58	12.09	10.34	9,1
CMV serology IGM (Ndx)	<0.9	-	1.5	-	-
CMV serology IGG (U/L)	<0.8	-	0,4	-	-
Nasopharyngeal swab	negative	negative	-	-	-

ALP = alkaline phosphatase; ALT = alanine aminotransferase; AST = aspartate aminotransferase; CRP hs= C-reactive protein hs; CMV = cytomegalovirus; GGT= Gamma Glutamyl Transferase; LDH= lactate dehydrogenase; NT- pro BNP = N-terminal pro brain natriuretic peptide; WBC = white blood cell count.

**Figure 5:**
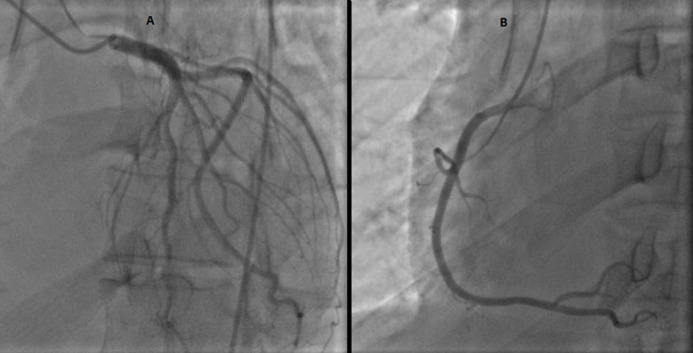
coronary angiography; revealing no obstructive atherosclerosis lesions in the coronary arteries

**Figure 6:**
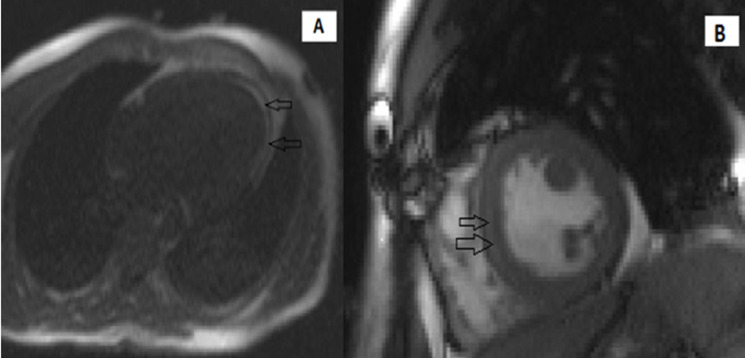
MR imaging findings: (A) horizontal long axis true-FISP performed after gadolinium infusion demonstrated a subepicardial enhancement (arrows); (B) short-axis showing delayed-enhancement imaging (arrows). True-FISP = True Fat Imaging with steady-state precession

Coronary angiography revealed no obstructive atherosclerosis lesions in the coronary artery (Figure 5). Most of the etiologic analyses for this dilated cardiomyopathy remained negative. ELISA tests demonstrated a recent CMV infection (IgG negative, IgM positive) (Table 1). An extensive biological screening was performed to identify another potential cause of acute hepatitis: total IgG was negative. We ruled out drug induced and autoimmune hepatitis, hereditary hemochromatosis and 1-antitrypsin deficiency. Abdominal ultrasonography showed normal liver characteristics with no biliary tract dilatation. Cardiac MR-scan was performed Day 6. Delayed enhancement sequences demonstrated subepicardial enhancement in the lateral free-wall of the left ventricle (Figure 6). The diagnosis of CMV-induced fulminant myocarditis was made on the basis of the acute clinical presentation and serologic evidence of infection. His hepatitis and pulmonary lesions were probably due to this primary CMV infection. The cardiogenic shock and the congestive heart failure were treated with intravenous inotropic agents and diuretic; without the need of an anti-viral medication. Two days later, the patient showed clinical improvement. We started supportive medication that included oral administration of 5 mg of ramipril, 2.5 mg of bisoprolol, 40 mg of furosemide twice a day, and 25 mg of spironolactone once a day. During the next week, the patient made steady progress, his liver enzyme levels and infection-related markers decreased (Table 1). After 13 days of hospitalization, the electrocardiogram, chest CT and echocardiography had become normal (Figure 2B, Figure 3B and Figure 4B). He was discharged under bisoprolol 2.5 mg and ramipril 5 mg per day. The patient remained asymptomatic with no further episodes of breathlessness at two-week follow-up.

## Discussion

The coronavirus disease 2019 pandemic forced tremendous changes in the functioning of the healthcare systems of all countries. The most common symptoms of COVID-19 are: fever, cough, Myalgia and dyspnea [1]. The most common laboratory abnormalities in COVID-19 are elevated serum C-reactive protein, lactate dehydrogenase, ferritin levels and D-dimer concentrations [1]. Elevated concentrations transaminases, or total bilirubin have been rarely reported [2]. About 20% of patients with COVID-19 develop cardiac injury with elevated N-terminal pro-B-type natriuretic peptide concentration, and increased cardiac troponin level [1]. One of the life-threatening cardiac manifestations is coronavirus fulminant myocarditis [3]. Our patient had all the clinical and biological features above. This made us highly suspect a COVID-19. Two nasopharyngeal swab were performed and turned out to be negative for COVID-19. Given the possibility of false negative test, healthcare workers continue to take extreme precautions. This impacted his management and limited access to routine cardiac diagnostics, such as MR- scan. AS for CMV myocarditis, it has been shown in immunocompetent adults in about 18 case reports per literature review [4]. The presentation can range from normal ECG or echocardiography [5] to cardiac enzymes elevation, heart failure and cardiogenic shock as we found in this case. A presumptive diagnosis of CMV myocarditis is made on the basis of high CMV IgM titers, cardiac biomarker elevation, abnormal echocardiogram and MR- scan as we did in this case.

Abnormalities based on liver function tests are often reported in immunocompetent adults with clinically significant primary CMV infection [5]. Alanine transaminase levels are more affected than the aspartate transaminase levels. Few weeks later, we usually have a progressive normalization of transaminase levels [5,6]. In this case, transaminase increased significantly as well as bilirubin levels, whereas GGT and ALP levels remained normal. The data regarding the need for specific antiviral therapy in immunocompetent subjects with severe CMV infection, are conflicting [6,7]. Any presumed benefit derived from specific antiviral therapy must be weighed against its potential toxicity [6]. No conclusions can be drawn about the indication of anti-viral therapy from the few experiences reported in literature [8]. In the present case, we chose not to provide ganciclovir therapy. Satisfactory progress was seen with total recovery of ventricular function and decreased dilation of the cardiac chambers, improvement of respiratory, and liver manifestations spontaneously. An open-label, pilot, randomised trial studied the withdrawal of pharmacological heart failure treatment in patients with recovered dilated cardiomyopathy and concluded that it was associated with relapse in 40% of cases [9]. The patients with previous episode of myocarditis represented only a small part in the 18% (9 patients) with dilated cardiomyopathy secondary to a trigger. The questions of whether we should keep pharmalogical treatment of heart failure after the recovery of dilated cardiomyopathy caused by myocarditis, and for how long, remain to be answered. Convincing a patient whose symptoms and cardiac function have disappeared to keep the treatment can be difficult.

## Conclusion

During the COVID-19 pandemic patients may develop myocarditis and heart failure, still every myocarditis is not a COVID-19 infection. CMV should always be considered a potential causative agent of severe myocarditis, even in an immunocompetent host. The present case illustrates that antiviral therapy is not always necessary

## References

[1] Sławiński G, Lewicka E (2020). What should a cardiologist know about coronavirus disease 2019 Kardiol Pol. Europe PMC.

[2] Lippi G, Plebani M (2020). Laboratory abnormalities in patients with COVID-19 infection. Clin Chem Lab Med.

[3] Zeng JH, Liu YX, Yuan J, Wang F, Wu W, Li J (2020). First case of COVID-19 complicated with fulminant myocarditis: a case report and insights. Infection.

[4] Santosh KP, Anupam K, Sandeep P (2014). Fulminant Cytomegalovirus Myocarditis in an Immunocompetent Host: Resolution with Oral Valganciclovir. Tex Heart Inst J.

[5] Hurt C, Tammaro D (2007). Diagnostic evaluation of mononucleosis-like illnesses. Am J Med.

[6] Bonnet F, Neau D, Viallard JF, Morlat P, Ragnaud JM, Dupon M (2001). Clinical and laboratory findings of cytomegalovirus infection in 115 hospitalized non immunocompromised adults. Ann Med Interne (Paris).

[7] Zubiaurre L, Zapata E, Bujanda L, Castillo M, Oyarzabal I, Gutiérrez-Stampa MA (2007). Cytomegalovirus hepatitis and myopericarditis. World J Gastroenterol.

[8] Siegal DS, Hamid N, Cunha BA (2005). Cytomegalovirus colitis mimicking ischemic colitis in an immunocompetent host. Heart Lung.

[9] Brian PH, Rebecca W, Amrit SL, Khalique Z, Gregson J, Newsome S (2019). Withdrawal of pharmacological treatment for heart failure in patients with recovered dilated cardiomyopathy (TRED-HF): an open-label, pilot, randomised trial. Lancet.

